# Bacteria in Milk

**Published:** 1889-07

**Authors:** 


### Bacteria in Milk.

The Berlin correspondent of the *Lancet*, March 9, 1889, says that it is intended to establish a bacteriological laboratory for the investigation of milk. The idea is due mainly to Dr. Hueppe, of Wiesbaden, who has for several years made a special study of the bacteria that appear in milk. He has definitely proved that lactic acid fermentation is caused by a special fungus, and butyric acid fermentation by another. Prazmowski, Liborius, Fuchs, and Neelsen have discovered other bacteria in milk, and their purely scientific researches, undertaken solely with a view to widening the limits of bacteriology, are now bearing valuable practical fruit. It is known that infant mortality is due largely to unwholesome milk, which can now be rendered harmless by sterilization. Further practical advantages are certain sooner or later to accrue from these investigations.

Treatment of Burns.—Nikolsky claims to have had excellent success in the treatment of burns of the second degree with fomentations of the following: Tannin, alcohol (95) aa 4 grammes, rectified sulphuric ether 30 grammes. The dressings are repeated two or three times a day. After the ether evaporates there remains a fine pellicle of tannin that causes prompt sedation of the pain and inflammation. The cure takes place much more rapidly than under the employment of collodion and other remedies in common use. The first dressing should be preceded by an antiseptic washing of the affected parts. The bullæ are punctured to allow the serum to escape, and the denuded surfaces are then powdered over with iodoform in a thin layer, and then dressed with the ether-tannin.—*Journal de Medicine de Paris*, May 20, 1888.

